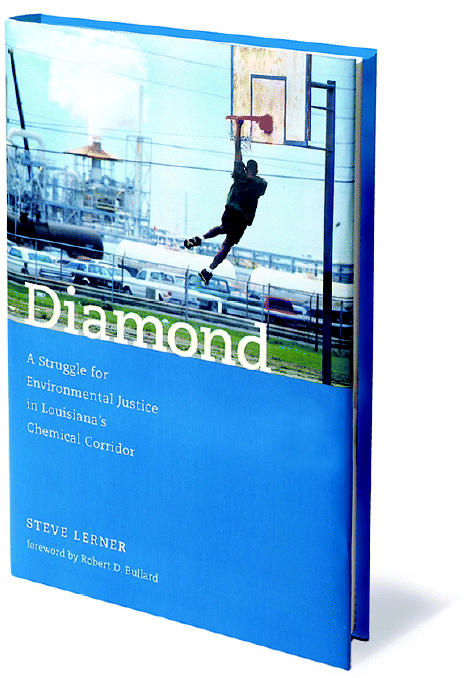# Diamond: A Struggle for Environmental Justice in Louisiana’s Chemical Corridor

**Published:** 2005-01

**Authors:** Dan VanderMeer

**Affiliations:** Dan VanderMeer was among the senior management staff at the Centers for Disease Control and Prevention that created its Center for Environmental Health. In 1984 he received the U.S. Public Health Service highest civilian award for helping establish the federal Agency for Toxic Substances and Disease Registry. He was the Department of Health and Human Services’ principal representative in the response to the national disaster at Love Canal where he coordinated health studies and recommendations in support of the eventual relocation and restoration of a major portion of the community.

By Steve Lerner

Cambridge, MA:MIT Press, 2004. 296 pp. ISBN: 0-262-12273-1, $27.95 cloth

Amazon.com lists over a dozen volumes on environmental justice, but only a few recent writings about disproportionately higher exposures to toxic and hazardous substances in low-income communities and people of color. Now there is another.

Lerner and others writing on this topic make it clear that “environmental justice” is an oxymoron. As a rule, those who live and work where chemicals and radioactive materials are produced are poor. Likewise, wastes from homes, industries, and weapons manufacture are deposited in poor communities. Environmental injustice is perhaps a better descriptor.

Accusations of environmental racism are not uncommon among those living in affected communities and their advocates. This was true in Diamond, a small, exclusively African-American neighborhood in the town of Norco, a few miles upriver from New Orleans. Norco was named for the New Orleans Refining Company built on plantation land. Over time, the oil business expanded to produce fuels, solvents, and other petroleum-based substances. Royal Dutch Shell and its successor, the Motiva-Equilon Alliance, have owned the business in recent decades. Lerner details this history, describing how complex social, racial, and economic factors combined to deny African-American residents many potential benefits of the growth of the oil industry. He lays out the conditions that led them to believe passionately that their health and well-being were at severe risk from exposures to pollution released from the refinery.

A major explosion occurred in a unit in the Shell refinery early morning on 4 May 1988. Seven workers died and 48 people were injured, some of whom were community residents. Over 4,000 people were temporarily evacuated. Lerner suggests that this was a tipping point for the Diamond community. The event lurked in the background as data describing leaks of pollutants into the surrounding areas were reported by Shell in the 1990s in response to new federal rules. Leadership in the community evolved. Margie Richardson, a local teacher, formed Concerned Citizens of Norco. Environmental justice advocates gravitated to Norco to support the citizens. And eventually Shell agreed to buy out the neighborhood. The book provides a deep level of detail on these people and the process.

The epic struggles in the 1970s and 1980s among residents, industry, and government to find a satisfactory resolution to community exposures to hazardous substances all seem to have a curiously common set of characters: the key local community activist(s) who emerge(s) from obscurity to a position of leadership; a core of nonresident advocates who bolster these community leaders; a representative who speaks for the industry but cannot commit the fiscal resources for putting things right; local, state, and federal environmental and health agency representatives who can help define the problem but are powerless to change the fundamental conditions in the community; expert consultants retained by the community, government, and industry—few of whom agree and none of whom are trusted by a majority; attorneys representing citizens or industry; and, finally, the media reporting on key events and observers (usually academics) who write papers and books about the process.

These were all present in Diamond. Lerner identifies with citizens and advocates who demanded relocation. Unlike white residents of Norco, the African-American community perceived unacceptably high risks and insignificant benefits. Although they lived with the same environmental contaminants, white residents believed that the oil economy strengthened their economic, educational, and social infrastructure. They did not support their African-American neighbors’ demands to have their houses purchased by the oil industry, and they did not relocate. Lerner notes that the unity of purpose in the Diamond neighborhood eventually resulted in a buyout and exodus from a foul and potentially dangerous toxic chemical environment. This success also disbanded an African-American community with roots spanning three centuries and ended the de facto segregation of Norco.

## Figures and Tables

**Figure:**